# Synergistic Interfacial and Doping Engineering of Heterostructured NiCo(OH)_x_-Co_y_W as an Efficient Alkaline Hydrogen Evolution Electrocatalyst

**DOI:** 10.1007/s40820-021-00639-x

**Published:** 2021-05-03

**Authors:** Ruopeng Li, Hao Xu, Peixia Yang, Dan Wang, Yun Li, Lihui Xiao, Xiangyu Lu, Bo Wang, Jinqiu Zhang, Maozhong An

**Affiliations:** MIIT Key Laboratory of Critical Materials Technology for New Energy Conversion and Storage, School of Chemistry and Chemical Engineering, Harbin Institute of Technology, Harbin, 150001 People’s Republic of China

**Keywords:** Interfacial and doping engineering, Heterostructured electrocatalyst, Solar-driven, Hydrogen evolution, Urea-assisted water splitting

## Abstract

To achieve high efficiency of water electrolysis to produce hydrogen (H_2_), developing non-noble metal-based catalysts with considerable performance have been considered as a crucial strategy, which is correlated with both the interphase properties and multi-metal synergistic effects. Herein, as a proof of concept, a delicate NiCo(OH)_x_-Co_y_W catalyst with a bush-like heterostructure was realized via gas-template-assisted electrodeposition, followed by an electrochemical etching-growth process, which ensured a high active area and fast gas release kinetics for a superior hydrogen evolution reaction, with an overpotential of 21 and 139 mV at 10 and 500 mA cm^−2^, respectively. Physical and electrochemical analyses demonstrated that the synergistic effect of the NiCo(OH)_x_/Co_y_W heterogeneous interface resulted in favorable electron redistribution and faster electron transfer efficiency. The amorphous NiCo(OH)_x_ strengthened the water dissociation step, and metal phase of CoW provided sufficient sites for moderate H immediate adsorption/H_2_ desorption. In addition, NiCo(OH)_x_-Co_y_W exhibited desirable urea oxidation reaction activity for matching H_2_ generation with a low voltage of 1.51 V at 50 mA cm^−2^. More importantly, the synthesis and testing of the NiCo(OH)_x_-Co_y_W catalyst in this study were all solar-powered, suggesting a promising environmentally friendly process for practical applications.

**Supplementary Information:**

The online version contains supplementary material available at 10.1007/s40820-021-00639-x.

## Highlights

- A promising solar-powered environmentally friendly process for the synthesis and application of catalysts for hydrogen evolution reaction has been proposed.
- A delicate NiCo(OH)_x_-Co_y_W catalyst with a bush-like heterostructure was realized via gas-template-assisted electrodeposition, followed by electrochemical etching process.
- The excellent catalytic effect of NiCo(OH)_x_-Co_y_W for the hydrogen evolution reaction was systematically investigated through various physical and electrochemical analyses.

**Supplementary Information:**

The online version contains supplementary material available at 10.1007/s40820-021-00639-x.

## Graphical abstract

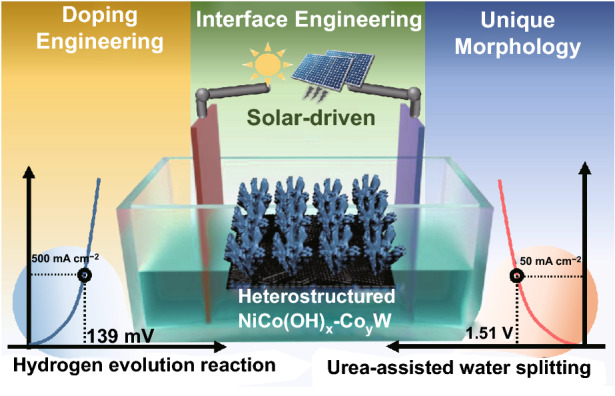

## Introduction

Hydrogen energy is considered the preferred alternative to fossil fuels for satisfying the huge global energy demands as it is environmentally friendly, renewable and has ultra-high energy density. Currently, alkaline water splitting (AWS) electrolyzers are still the most reliable among the many large-scale hydrogen (H_2_) production strategies [1–3]. As the core half-reaction, the electrocatalytic performance of the hydrogen evolution reaction (HER) should be given priority with an ideal low potential and high working current density, which can be achieved by virtue of the excellent activity of electrocatalysts to overcome the limitation of sluggish kinetics [4–8].

Further, 2D transition metal-based (Ni, Co, Fe, Mo, W, and Mn, among others) catalysts (TMCs) exhibiting remarkable activity have emerged as mainstream strategies to support effective HER [5, 9–12]. Among them, Ni- and Co-based hydroxides have been reported as exhibiting promising HER activity through motivated H_2_O absorption and strengthened OH^−^ desorption, thereby enhancing the alkaline water dissociation kinetics (Volmer step) of HER [13–15]. Although the synergistic effect between polymetallic atoms can be targeted to change the electronic structure and activate H_2_O absorption sites, the poor charge transport, insufficient H* sites, and excessive adsorption energy limit individual performance. Bearing the correlation between catalytic behavior and interface properties, interface engineering is a priority strategy, and the construction of an M(OH)_x_-M (M: metal) heterostructure is a representative strategy for enhancing HER activity, even for bifunctionality [16–20]. Many kinds of metals (Pt, Pd, Ni, Co, and NiCo, among others) can be effectively coordinated with hydroxides to break through the catalytic performance [21–25]. Emphatically, intermetallic tungsten bonding with other transition metals is regarded as a candidate worth consider because of the more moderate binding energy resulting from band structure changes, as well as greatly increased edge sites, by activating the close-packed basal surface in the metal phase [26, 27]. Some recent studies have proven that the strong interaction between Ni and W was beneficial for achieving improved HER performance [28–30]. Moreover, some similar groups of compounds, such as Ni-Mo and Co-Mo, have also been reported with optimized cyclitic effects [31–33]. Therefore, it is not hard to expect that the effective combination of Co-W might also have an analogous favorable influence, and developing a W-doped metallic solid solution + Ni-based hydroxide polymetallic heterostructure with good morphology is valuable for motivating alkaline HER activity.

Conversely, the sluggish kinetics of the oxygen evolution reaction (OER, 1.23 V vs. RHE) restrict the efficiency of the overall electrolyzer to some extent, and adopting the urea oxidation reaction (UOR) with a lower theoretical potential (0.37 V vs. RHE) to replace the OER would effectively upgrade the overall electrocatalytic performance [34–36].

Enlightened by the above discussions, a Janus-like, delicate NiCo(OH)_x_-Co_y_W catalyst with a bush-like heterostructure was fabricated on carbon paper (CP) via gas-template-assisted electrodeposition, followed by an electrochemical etching-growth process. As expected, owing to the polymetallic synergy and heterogeneous structures, NiCo(OH)_x_-Co_y_W exhibits remarkable HER performance in an alkaline solution, with an ultralow overpotential of 21 and 139 mV to deliver 10 and 500 mA cm^−2^, respectively. Further in-depth analysis suggests that (1) the 2D nanosheet + 3D bush-like structure provides sufficient surface area and an adequate bubble diffusion path; (2) integrating NiCo hydroxide with a CoW solid solution results in electron redistribution at the interface, which is associated with the metallicity together promoting electrical conductivity and mass transfer; (3) amorphous NiCo hydroxide serves to accelerate the dissociation barrier of water molecules (Volmer step); (4) defect enrichment in the W-doped phase is employed to provide sufficient sites, moderate binding energy for adsorption/desorption of the H intermediate, and H_2_ release (Heyrovsky step and Tafel step). In addition, NiCo(OH)_x_-Co_y_W also performs well in UOR and is encouraged through bifunctionality. A two-electrode electrolyzer (1 M KOH + 0.3 M urea) is used to drive overall electrolysis at a low potential of 1.51 V to deliver 50 mA cm^−2^. Importantly, the energy efficiency of the catalyst synthesis and test was realized by a solar-powered system. This work provides an insight into the design and effective synthesis of heterostructured and intermetallic catalysts via a novel route, while suggesting a very promising environmentally friendly process for practical applications.

## Experimental Section

### Chemicals

CoSO_4_·6H_2_O (≥ 99.0%), Na_2_WO_4_ (≥ 99.0%), Na_3_(C_3_H_5_O_7_)·2H_2_O (≥ 99.0%), NiCl_2_·2H_2_O (≥ 99.0%), NaCl (≥ 99.0%), urea (≥ 99.0%), Nafion solution (5 wt%), platinum on carbon (20 wt% Pt/C), KOH (≥ 99.0%), and hydrochloric acid (HCl, ≥ 99.0%) were used as received, without further purification, and all aqueous solutions were prepared with ultrapure water (> 18.25 MΩ cm) obtained from Millipore system.

### Synthesis of CoW and NiCo(OH)_x_-Co_y_W

The electrodeposited CoW solid solution was executed with a two-electrode system, 100 mL electrolyte contained CoSO_4_·6H_2_O (150 mmol), Na_3_(C_3_H_5_O_7_)·2H_2_O (90 mmol), Na_2_WO_4_·2H_2_O (25 mmol), and Na_2_SO_4_ (5 mmol), with an adjustment pH value of 5.5. CP and graphite rods were used as the working and counter electrodes, respectively. An optional reaction potential of − 8 V was held for 800 s at ambient temperature and powered via a solar-photovoltaic electricity system. The as-prepared sample was then transferred into an etching solution (NiCl_2_·6H_2_O + NaCl) for 12 h to achieve the internal redox reaction and spontaneous growth of NiCo hydroxide. All the compared samples were treated with the same process, and the Cl^−^ and Ni^2+^ contents were adjusted.

### Electrochemical Measurement

The electrochemical measurements were performed using a CHI 660e Electrochemistry Workstation. In the three-electrode measurement system, a graphite rod and saturated calomel electrode were adopted as the counter electrode and reference electrode, respectively. All as-prepared samples were directly used as the working electrodes. The overall splitting test was conducted using NiCo(OH)_x_-Co_y_W for both the anode and cathode. The HER measured solution was 1.0 M KOH solution (pH = 13.7, 50 mL), and 1 M KOH + 0.3 M urea solution was used in UOR and urea-assisted water splitting. Linear sweep voltammetry (LSV) polarization curves were acquired at a scan rate of 5 mV s^−1^ with 85% IR compensation. Electrochemical impedance spectroscopy (EIS) measurements were performed at frequencies ranging from 10 to 0.001 Hz at − 0.1 V (vs. RHE). The double-layered capacitances (*C*_dl_) of these fabricated electrodes were measured by cyclic voltammetry (CV) curves from 0.11 to 0.21 V (vs. RHE) for HER at different scan rates, and ECSA was estimated by the value of *C*_dl_/0.04.

### Characterization

The morphology was characterized via scanning electron microscopy (SEM, Hitachi S4800, 5 kV), atomic force microscopy (AFM, Dimension Fastscan), and transmission electron microscopy (TEM, JEM 2100, 200 kV). X-ray diffraction (XRD, D/max2550V with Cu Kα radiation of *λ* = 1.541841 Å) was used to determine the crystal structure. The surface composition and valence of the sample were analyzed using X-ray photoelectron spectroscopy (XPS, Thermo Escalab 250, C 1 s peak at 284.5 eV, and monochromatic He I light source of 21.2 eV). Fourier transform infrared (FT-IR) spectroscopy was used to monitor the functional group distribution on the material surface. Scanning Kelvin probe (SKP) measurement (SKP5050 system, Scotland) was adopted to detect work function in an ambient atmosphere, and a gold electrode was used as the reference.

## Results and Discussion

### DFT Analysis for the Effect of Tungsten Introduction

**Fig. 1:**
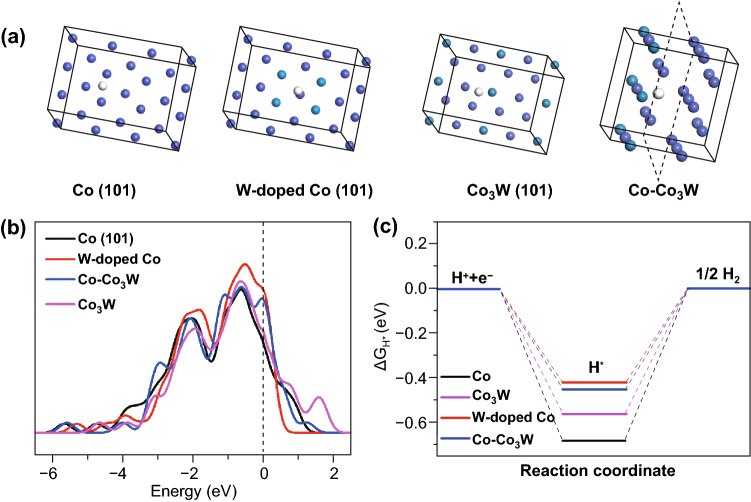
**a** Top (up) views of the Co, W-doped Co, Co_3_W and Co-Co_3_W heterostructure. **b** Density of states comparison with or without W introduction. **c** Free energy diagrams for H* adsorption

Modifying the adsorption free energy of *H*_ads_ (Δ*G* *H*_ads_) is undoubtedly the key factor that accelerates the reaction kinetics of the Heyrovsky step or Tafel step, thus activating the HER activity. A previous study suggested that metallic Co has extreme adsorption that restricts H_2_ formation, and a feasibility strategy involved downshifting the d-band center via cation doping to adjust the Δ*G* *H*_ads_ [37, 38]. Thus, density functional theory (DFT) calculations were performed to determine the HER adsorption mechanism of CoW. The optimized models of Co with or without W doping, Co_3_W, and heterostructured Co-Co_3_W are shown in Fig. 1a. The charge density distribution visually demonstrates the difference between the tungsten and cobalt atoms (Fig. S1). The introduction of W effectively regulates the electronic environment in the form of doping or a new phase, thus tuning the d-band center toward the Fermi level (Fig. 1b). W-doped Co, Co_3_W, and Co-Co_3_W exhibit a more appropriate ΔG H_ads_ value of − 0.42, 0.55, and 0.45 eV, i.e., higher than the Co site without introduction of W (− 0.69 eV) (Fig. 1c). The DFT results indicate that the collaborative Co and W sites, and the Co-Co_3_W interface are beneficial for the adsorption/desorption kinetics of H_ads_ with the local electronic redistribution, suggesting the synergistic effect of Co-W collaboration.

### Structure and Composition Characteristics

**Fig. 2:**
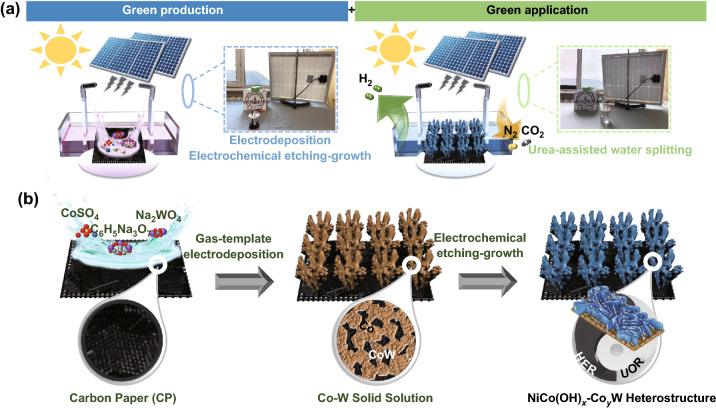
**a** Schematic of green production of catalyst and green application of hydrogen evolution. **b** Illustration of the synthetic process for NiCo(OH)_x_-Co_y_W

**Fig. 3:**
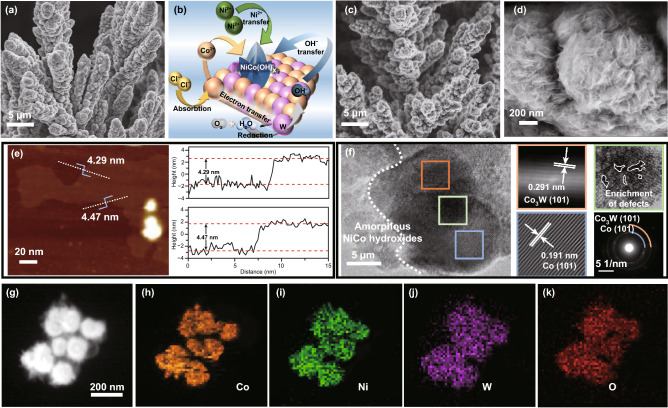
**a** SEM image of CoW. **b** Illustration of the etching-growth mechanism and **c**, **d** SEM image of CoW-500-Ni with different magnification. **e** AFM image. **f** HRTEM image, partial Fourier transform and SAED image. **g**, **h** HAADF-TEM image and elemental mappings of CoW-500-Ni

Figure 2a shows a schematic of an environmentally friendly process for this study. The synthesis and use of NiCo(OH)_x_-Co_y_W were all solar-driven processes with zero-pollution. The detailed preparation of NiCo(OH)_x_-Co_y_W catalyst is shown in Fig. 2b. The precursor of CoW solid solution (labeled CoW) supported on CP (1 × 1 cm^−2^) was first prepared by solar-driven gas-template electrodeposition, followed by soaking in the etching solution (NiCl_2_·6H_2_O + NaCl) to form an in situ external NiCo hydroxide shell. The as-prepared CoW solid solution underwent a deposition at different potentials for 800 s. The inevitable H_2_ evolution occurs simultaneously with deposition, which can be exploited as a dynamic template that prompts upward vertical growth (Movie S1 and Fig. S2). SEM images of Figs. 3a and S5 show that under a potential of − 8 V, the bush-like CoW is arranged at a high density and enriched with porous structure. For comparison, the other samples deposited at − 1.5 and − 5 V were also synthesized without the obvious longitudinal growth structure (Fig. S3), suggesting that the deposited potential is a key factor for the final obtained production morphology (Fig. S4). Then, the CoW precursor was immersed in an etching solution (50 mM NaOH and 500 mM NaCl) to undergo surface reconstruction via electrochemical corrosion (labeled as CoW-500-Ni). The growth mechanism is illustrated in Fig. 3b. The oxygen corrosion reaction consists of an oxygen reduction reaction (O_2_ → OH^−^), while the adsorbed Cl^−^ ion also drives the electron transfer process. A higher cobalt content could take precedence over tungsten to participate in metal oxidation behavior (Co + nOH^−^ → Co(OH)_x_ + ne). Regarding the effect of immersion time, it was observed that no significant hydroxide nanosheet morphology initially emerged. With increased immersion time, an ideal morphology of hydroxide nanosheet array can be obtained after 12 h, while prolonged time causes the active material to partially fall off (Fig. S6), suggesting that immersion time is an important factor in the final obtained morphology. SEM images with different magnifications in Fig. 3c and d show that for CoW-500-Ni**,** the soaking treatment maintains the overall structural integrity with array coating. The Brunauer–Emmett–Teller (BET) specific surface area of CoW-500-Ni was calculated as 1.93 m^2^ g^−1^ (Fig. S7). The ultrathin hydroxide nanosheet in the coating arrays was approximately 4.36 nm, as measured by atomic force microscope (AFM, Fig. 3e), ensuring an abundant surface area for the reaction contact. In Fig. 3f, high-resolution transmission electron microscopy (HRTEM) images show that the interface intuitively exists between the metallic phase and the amorphous hydroxide phase. The well-defined lattice fringes with inter-planar distances of 0.291 and 0.191 nm corresponding to Co (101) and Co_3_W (101), respectively, specify the formation of Co and CoW alloy, as further analyzed by selected area electron diffraction (SAED). In addition, the presence of observable internal defects within a solid solution is considered beneficial for providing abundant sites [33]. The homogeneous distribution of Ni, Co, W, and O was further demonstrated by TEM energy-dispersive X-ray spectroscopy (TEM-EDS) elemental mapping (Fig. 3g–j). The above characterizations confirm the successful construction of the NiCo(OH)_x_-Co_y_W catalyst with a hierarchical bush-like heterostructure.

**Fig. 4:**
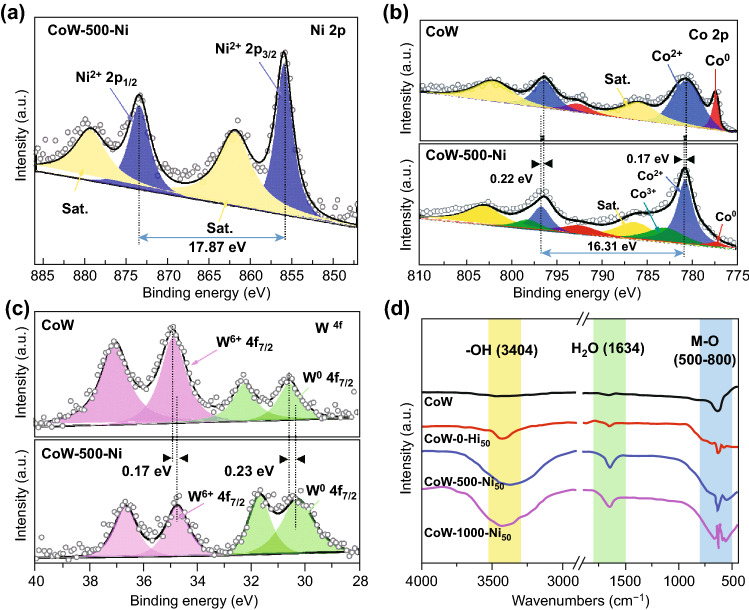
**a** Ni 2p XPS spectra of CoW-500-Ni. **b** Co 2p spectra and **c** W 4d spectra of the comparison between CoW-500-Ni and CoW. **d** FT-IR spectra of CoW and all the etching samples

XPS was further performed to clarify the chemical composition and coordinated electronic environment. The wide range spectrum of CoW indicates the coexistence of Co, W, and O elements, with atomic ratios of 74.2%, 11.9%, and 13.9%, respectively, which slightly different from the ICP result (Table S1); this is mainly due to the surface layer distribution of Ni. The increased O element ratio of CoW-500-Ni (Fig. S8) suggests the successful introduction of Ni. Detailed information corresponding to the peaks is presented in Table S2. In Fig. 4a, the Ni 2p high-resolution spectra of CoW-500-Ni exhibit two main peaks at 855.8 and 873.4 eV, corresponding to Ni 2p_3/2_ and Ni 2p_1/3_, respectively, with an energy gap of 17.6 eV, indicating the presence of Ni(OH)_2_ [39]. The Co 2p spectra of CoW and CoW-500-Ni are presented in Fig. 4b. For CoW, the peak at 777.3 eV corresponds to the Co^0^, and the peak at 780.6 eV corresponding to Co^2+^ results from the inevitable oxidation. Significantly in CoW-500-Ni, a new peak at 783.2 eV is attributed to the higher valence states of Co (Co^3+^) and the characteristic peaks of Co^0^ have little attenuation, which is attributed to the surface etching-growth process. The Co^2+^ peaks at 780.7 and 796.1 eV matched with the energy of Co–O also positively shift to 0.22 and 0.17 eV owing to the interfacial electron excursion [40]. In Fig. 4c, the binding energy of the W 4d peak in CoW can be deconvoluted into W^0^ (30.6 eV) and W^6+^ (34.7 eV) [40]. For CoW-500-Ni, the increased peak intensity ratios of W^0^ and W^6+^ with a negative shift (0.23 and 0.17 eV) were observed, indicating the extra charge transfer from the hydroxide to the internal metallic phase. The interfacial electronic redistribution between NiCo hydroxides and CoW is beneficial for activating the electrocatalytic properties [41].

Considering the criticality of Cl^−^ ions in etching, solutions with different NaCl concentrations (0 and 1000 mM) were also employed to investigate the impact systemically (labeled CoW-0-Ni and CoW-1000-Ni, respectively). The SEM images (Fig. S9) of the above two samples show that with the increase in Cl- concentration, the nanosheets become larger in size and more densely packed. Moreover, the same composition as CoW-500-Ni and an increase in O ratio were detected via XPS analysis (Fig. S10). To explore the influence mechanism of Cl^−^ concentration, the corrosion curve of the precursor (Fig. S11) was measured. The anodic corrosion potential of − 0.6 V (vs. SCE) is much lower than the O_2_ reduction potential, ensuring a spontaneous corrosion reaction, while the corrosion current density is positively correlated with Cl^−^ concentration. XRD patterns were then investigated for the crystalline characterization of the CoW solid solution, as shown in Fig. S12. Except for a wild peak at 21° of CP, the diffraction peaks match well with the standard pattern of Co (JCPDF No. 05-0727), certifying the poor crystallinity, and no significant characteristic peak was detected possibly due to the presence of a low quantity of W-doped alloy [42]. For the etched sample, the undetectable characteristic peaks of Ni and Co-based hydroxides, demonstrating their amorphous state, were consistent with the HRTEM analysis. FT-IR was utilized to test the surface changes before and after the soaking treatment. As shown in Fig. 4d, the bending vibration peaks at 3404, 1634 cm^−1^ and ranging in 500–800 cm^−1^ are belong to –OH, absorbed H_2_O and M–O (M: Ni and Co) respectively, further proving the generation of hydroxides [43]. For CoW and all of the etched samples, the increased peak intensity is positively correlated with the change of in Cl- concentration, which is attributed to the strengthened etching-growth behavior. Especially, no W–O bending vibration was detected at 832 cm^−1^, illustrating that W is not involved in surface reconstruction, and all echoes the above analysis [44]. These results not only reveal the successful construction of a mixed-crystalline polymetallic hydroxide heterostructure of NiCo(OH)_x_-Co_y_W but also confirm the significant interfacial effects, providing an encouraging opportunity for optimizing the catalytic performance.

### Electrocatalytic Performance

**Fig. 5:**
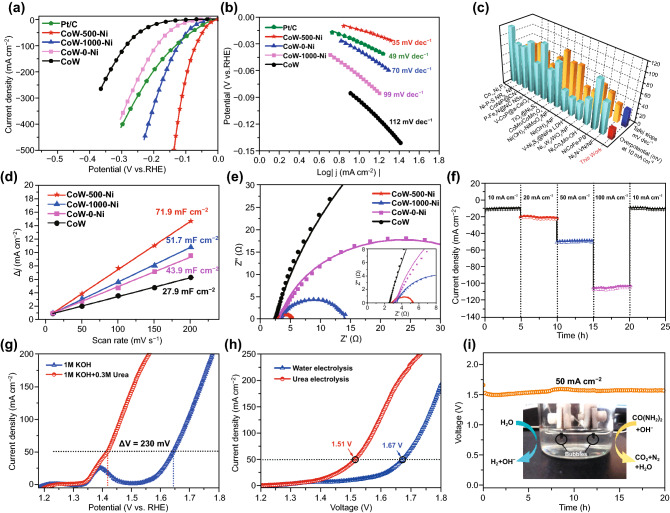
**a** HER polarization curves and **b** Tafel slopes of Pt/C, CoW, CoW-0-Ni, CoW-500-Ni, and CoW-1000-Ni. **c** Comparison of HER performance with recent represent work. **d** *C*_dl_ value and **e** EIS Nyquist plots at a given potential for different electrocatalysts. **f** Chronopotentiometric curves at different current densities. **g** UOR and OER polarization curves of CoW-500-Ni. **h** Overall splitting of CoW-500-Ni with and without urea assistant. **i** Galvanostatic curve of the urea-added electrolyzer at 50 mA cm^−2^

To achieve efficient hydrogen evolution, the electrocatalytic HER performance was first evaluated via LSV. Concurrently, the as-prepared samples (CoW, CoW-0-Ni, CoW-500-Ni, CoW-1000-Ni) and 20% Pt/C catalyst (1 mg cm^−2^) were also adopted for comparison. A three-electrode system was employed, and the polarization curves are shown in Fig. 5a with 85% IR compensation. It is clear that compared with CoW, the HER activity after soaking treatment was significantly enhanced. CoW-500-Ni exhibits superior activity with an extremely low onset potential close to 0 V, and a small overpotential of 21 mV (to deliver current densities of 10 mA cm^−2^), which is parallel to commercial Pt/C (28 mV) and is better than CoW-1000-Ni (52 mV) and CoW-0-Ni (91 mV). Furthermore, CoW-500-Ni also undergoes a high current density of 500 mA cm^−2^ with a low overpotential of 139 mV. The HER intrinsic activities are determined by its reaction kinetics, which can be intuitively visualized via the derived Tafel plots (Fig. 5b). The Tafel slope of CoW-500-Ni is 35 mV dec^−1^, which is lower than that of Pt/C (49 mV dec^−1^), CoW-1000-Ni (70 mV dec^−1^), CoW-0-Ni (99 mV dec^−1^), and CoW (112 mV dec^−1^). All these results indicate that the catalytic process follows the Volmer–Hylovsky mechanism, and the assembly of the heterostructure accelerates the control of the rate-determined Volmer step. Notably, CoW-500-Ni demonstrated smooth and highly competitive results among the recent representative HER catalysts (Fig. 5c and Table S3).

Further tests were performed to explore the details of the outstanding performance. Adequate active areas are extremely important for HER performance. The electrochemical surface area (ECSA) was calculated to investigate the density of active sites by double-layer capacitance (*C*_dl_) in Fig. 5d via cyclic voltammetry CV curves (Fig. S13). The calculated *C*_dl_ values of CoW-1000-Ni and CoW-0-Ni are 51.7 and 43.9 mF cm^−2^, respectively, which are obviously higher than the 27.9 mF cm^−2^ of CoW, illustrating that the proper etching treatment could dramatically increase the number of exposed active sites. In particular, CoW-500-Ni has a maximum *C*_dl_ of 71.9 mF cm^−2^ and the ECSA normalized LSV curves of all prepared samples demonstrate that CoW-500-Ni also has outstanding intrinsic activity. The TOF values of CoW-500-Ni at an overpotential of 100 mV are 0.87 s^−1^, which is significantly higher than that of the other samples, demonstrating the more active site exposure (Fig. S14). To studies the electrode kinetics, EIS was measured with the Nyquist fitting curves (Fig. 5e) at an operating overpotential of 100 mV. The calculated values are systematically listed in Table S4. By comparing the charge transfer resistance (*R*_ct_), CoW-500-Ni has a minimum value of 2.54 Ω, demonstrating the fast electron transport during the HER process, which is indispensable for excellent HER performance, especially at high current densities. The corresponding Bode curves demonstrate a single electrochemical process of charge transfer (Fig. S15). All of the above results indicate that etching treatment to assemble the mixed-crystal heterostructure is essential for promoting HER activity, and CoW-500-Ni is the best candidate, derived from its appropriate surface modification. Further, CoW-1000-Ni exhibited a Faradaic efficiency (FE) of approximately 100% (Fig. S16), with excellent energy efficiency during the alkaline HER process. Since the long-term stability of the catalyst is a key parameter for evaluating the practical application value, CoW-1000-Ni was then measured by chronopotentiometric curves with a multi-step current density (10, 20, 50, and 100 mA cm^−2^). As shown in Fig. 5f, CoW-500-Ni exhibits strong stability and only a small attenuation after 25 h at 10 mA cm^−2^. The SEM images after the stability test (Fig. S17) show that the primary morphology is well maintained, implying its robust long-term stability, which is consistent with the subsequent XPS results after 3 h HER test. Further HER tests of CoW-500-Ni were also carried out in a neutral system (pH = 7), but failed to demonstrate good catalytic activity (Fig. S18).

To explore the electrocatalytic bifunctionality, the anodic performance was also investigated. The LSV curves of all groups exhibit poor OER activity (an overpotential higher than 300 mV under 50 mA cm^−2^, Fig. S19), which is a significant obstacle to applications in electrolysis. Considering the lower theoretical voltage of the UOR (0.37 V) compared to water oxidation (OER, 1.23 V), we further measured the performance of CoW-500-Ni for OER and UOR in 1 M KOH with and without 0.3 M urea, respectively. As exhibited in Fig. 5g, the LSV curves show that the CoW-500-Ni requires only 1.34 V (vs RHE) to deliver 10 mA cm^−2^ and requires 1.41 V at a current density of 50 mA cm^−2^, which is clearly lower than that of the OER, indicating that a faster reaction kinetics of the urea oxidation process is preferential. We also tested the HER performance of urea introduction and the negligible change in Fig. S20 proved that there was almost no effect on the cathode reaction. The overall UOR activity is better than that of the OER for all candidates. In particular, the enhanced UOR performance of all the soaking groups was better than that of the CoW, directly confirming the promoting effect of the 0.3 M heterostructure (Fig. S21). Comparing the LSV curve under different urea concentrations, it is determined to be good for driving UOR (Fig. S22). Inspired by the superior Janus performance, the self-supported CoW-500-Ni was simultaneously employed as both the anode and cathode to drive urea-assisted electrolysis. In Fig. 5h, the anodic current density in the urea-added solution sharply rises at 1.34 V and only requires 1.51 V to achieve a current density of 50 mA cm^−2^, which is obviously lower than that required by water electrolysis (1.67 V), indicating significant energy saving. As shown in Fig. 5i, a 20-h working measurement in urea electrocatalysis with negligible activity loss also exhibits long-term durability. A fully solvable solar-powered system was assembled with an output voltage of 1.50 V, and concurrently, gas evolution at both electrodes was clearly visible (Fig. S23 and Movie S2), realizing the efficient and flexible conversion between renewable energy sources. The electrochemical test results indicate the enhanced electrocatalytic performance of CoW-500-Ni for efficient H_2_ evolution.

### Electrocatalytic Mechanism Analysis

**Fig. 6:**
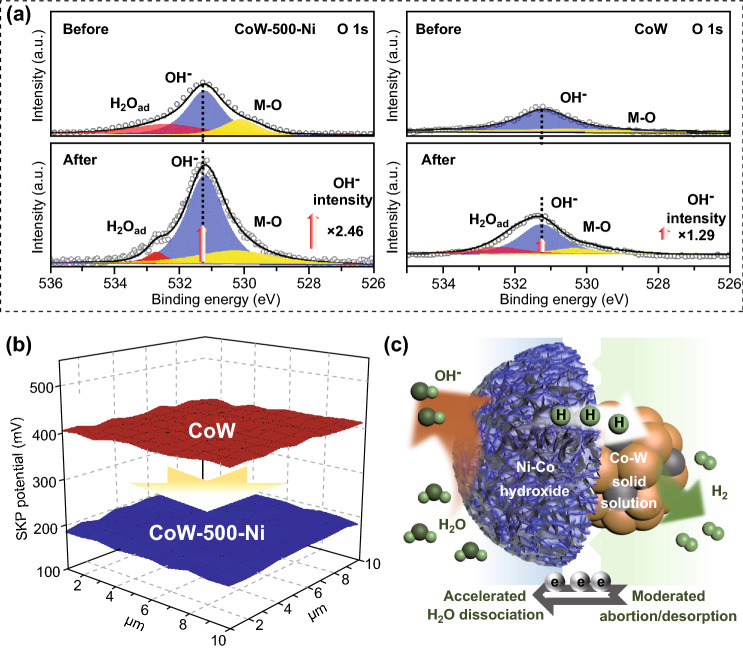
**a** XPS O 1s spectra of CoW-500-Ni and CoW before and after 3 h HER. **b** WF spectra comparison of CoW-500-Ni and CoW. **c** Schematic illustration of the optimal HER mechanism

On the basis of the remarkable electrocatalysis performance, we systemically characterized the heterostructured NiCo(OH)_x_-Co_y_W to determine the HER boost mechanism. First, the surface chemical change of CoW-500-Ni after 3 h of HER was investigated via XPS analysis. A wide range of spectra and Ni 2p, Co 2p, and W 4f spectra (Fig. S24) confirm the compositional stability. Focusing on the O 1 s spectra, the monitored peak at 530.1 eV corresponds to the M–O functional group. As shown in Fig. 6a, the 531.2 eV peak of CoW-500-Ni is ascribed to OH^−^, which increases significantly after the HER test, whereas the OH^−^ peak of CoW only has a modest promotion. This phenomenon results from the amorphous NiCo-hydroxides having a stronger tensile effect on -OH, effectively enhancing the water dissociation ability and optimizing the reaction kinetics of the Volmer step [45]. Considering the effect of Ni content on the HER activity, different amounts (0 and 100 mM of Ni^2+^, samples denoted as CoW-500-Ni_0_ and CoW-500-Ni_100_, respectively) were used in the etching solution to conduct the study. SEM images and EDS analysis in Fig. S25 show that an increased Ni concentration acts directly in the catalyst atomic ratio of Ni without causing obvious morphological changes, and the LSV curves in Fig. S26 demonstrate that CoW-500-Ni (with 50 mM of Ni) is superior to the others. These results demonstrate the importance of the appropriate Ni-Co ratio to obtain the best activity. Second, work functions (WF) of heterogeneous structures are measured via a scanning Kelvin probe technique (SKPT) to explore the interface effect. In Fig. 6b, the calculated WF value of CoW-500-Ni (4.79 eV) is lower than CoW (5.01 eV), suggesting sufficient highway has been established, as well as a faster charge transfer ability that stems from the wide heterogeneous interface range to overcome hydroxide weak conductivity. Third, given the aforementioned DFT analysis and the comparative experiment analysis without the introduction of tungsten (Fig. S27), the surface adsorption energy of the CoW phase tends to be moderate due to W doping, which is conducive to the subsequent chemical desorption/electrocatalytic desorption. The internal defects observed in HRTEM provide additional sites for atom absorption, thus optimizing the reaction path from H_ads_ to H_2_ [46]. Finally, the bush-like arrays constructed by the densely stacked metal nanoparticles and the ultrathin amorphous hydroxide shells provide sufficient active area and fast gas release pathways, endowing exposed highly active areas and faster gas release pipelines [47, 48]. Summarized by the above discussion, Fig. 6c graphically illustrates the optimized HER mechanism of the NiCo(OH)_x_-Co_y_W electrocatalyst. Water molecule dissociation of the Volmer step occurs on the amorphous hydroxide surface sites and the generated H_ads_ tend to migrate toward the internal site for the Heyrovsky step or Tafel step while rapid charge transfer ensures that the reaction is sustained. Meanwhile, the mechanism analysis of the interface and doping engineering is also applied to the promoted UOR kinetics through the cyclic transformation of M-OH and M-OOH, which supports the considerable UOR activity of NiCo(OH)_x_-Co_y_W [35, 49, 50].

## Conclusions

In summary, a delicate electrocatalyst of NiCo(OH)_x_-Co_y_W with a bush-like heterostructure was realized via a novel and environmentally friendly synthetic route. As expected, the self-supported catalyst exhibited excellent HER performance in alkaline solutions. The mixed-crystalline heterostructure results in electron redistribution and amorphous NiCo hydroxide accelerates the Volmer step rate while the crystal CoW phase ensures the matching of the Tafel and Heyrovsky steps, thus promoting reaction kinetics. Additionally, the unique morphology endows a higher specific active area and faster gas release, while a heterogeneous interface guarantees efficient charge transfer. The synergistic effects of all steps determine the excellent HER performance. Meanwhile, NiCo(OH)_x_-Co_y_W also exhibited remarkable UOR activity. Prompted by the bifunctional performance, it succeeded in both cathodic H_2_ generation and anodic urea oxidation under the drive of a solar—power system and attained a current density of 50 mA cm^−2^ at only 1.51 V. This work provides an innovative guidance for the design and synthesis of heterostructured catalysts, while also confirming the broad prospects of electrolysis hydrogen production in practical applications.

## Supplementary Information

Below is the link to the electronic supplementary material.

Supplementary file1 (MP4 1226 KB)

Supplementary file2 (MP4 1371 KB)

Supplementary file3 (docx 14590 KB)
