# Supplementary material for: Synergistic Interfacial and Doping Engineering of Heterostructured NiCo(OH)x-CoyW as an Efficient Alkaline Hydrogen Evolution Electrocatalyst
Source: Nanomicro Lett. 2021 May 3;13:120. doi: 10.1007/s40820-021-00639-x (PMC8093358; doi:10.1007/s40820-021-00639-x)
Supplement: Supplementary file 3 — Supplementary file3 (docx 14590 KB) [file 40820_2021_639_MOESM3_ESM.docx]

Supporting Information for

**Synergistic interfacial and doping engineering of heterostructured NiCo(OH)_x_-Co_y_W as an efficient alkaline hydrogen evolution electrocatalyst**

Ruopeng Li, Hao Xu, Peixia Yang^🖂^, Dan Wang, Yun Li, Lihui Xiao, Xiangyu Lu, Bo Wang^🖂^, Jinqiu Zhang, Maozhong An

🖂Peixia Yang, [yangpeixia@hit.edu.cn](mailto:yangpeixia@hit.edu.cn)

🖂Bo Wang, [wangbo19880804@163.com](mailto:wangbo19880804@163.com)

MIIT Key Laboratory of Critical Materials Technology for New Energy Conversion and Storage, School of Chemistry and Chemical Engineering, Harbin Institute of Technology, 150001 Harbin, China.

This profile includes **Computational details, 27 Figures, 3 Table, 15 references.**

**Computational details**

All the DFT calculations were performed through the Perdew–Burke–Ernzerhof (PBE) functional, which has the based group of double numerical atomic orbital plus polarization function (DNP), and carried out by D Mol3 module of Materials Studio Package under generalized gradient approximation. All the models used were based on the Co (101). The energy convergence clause, maximum force, as well as maximum displacement were programmed at 105 eV, 0.01 eV Å1 and 0.005 Å to realize the optimal geometry state. Applying 0.005 Ha is to ensure accurate electronic convergence.

**
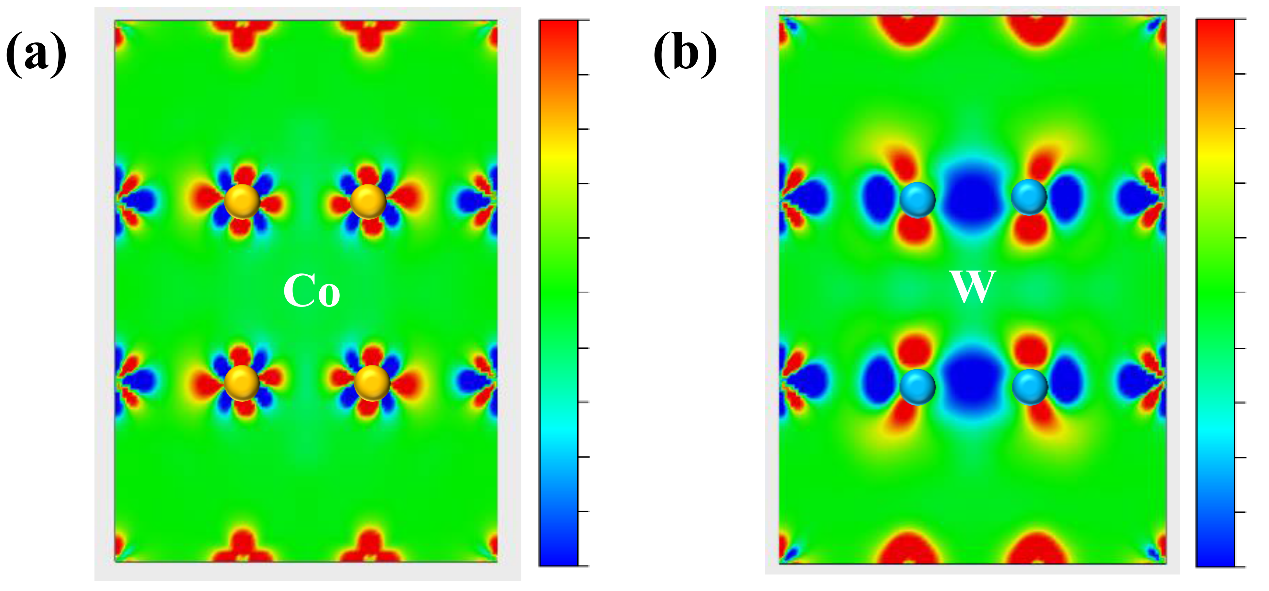
**

**Figure S1.** Charge density difference of a) Co and b) W.


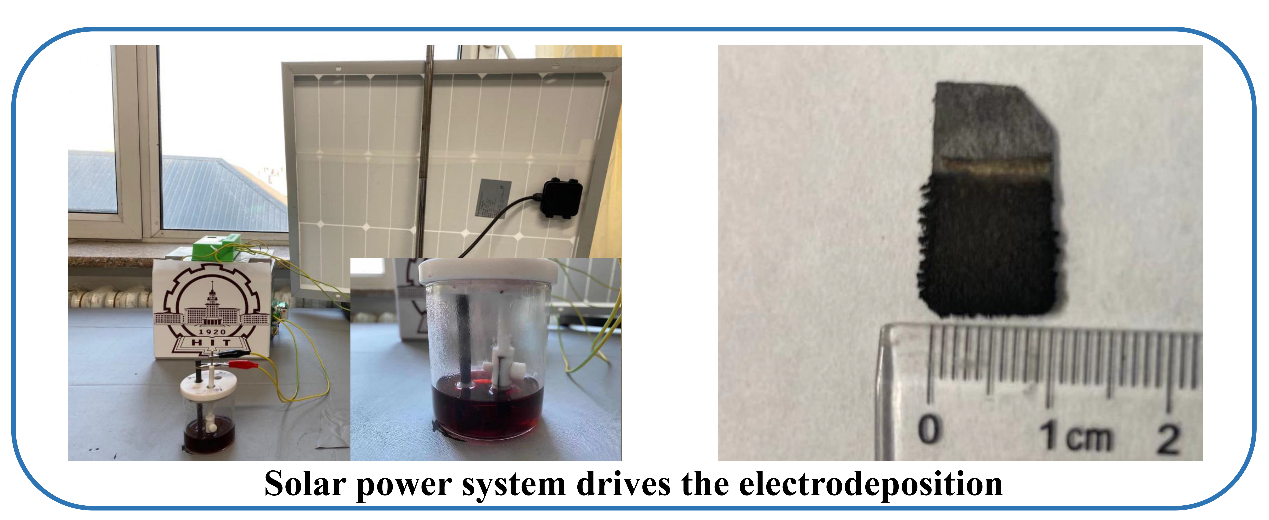


**Figure S2.** Left is the photographic image of the solar-driven electrodeposition system to prepared CoW electrode at the deposited potential of -8 V with 800s. Right is the fabricated CoW electrode growth on the carbon cloth.


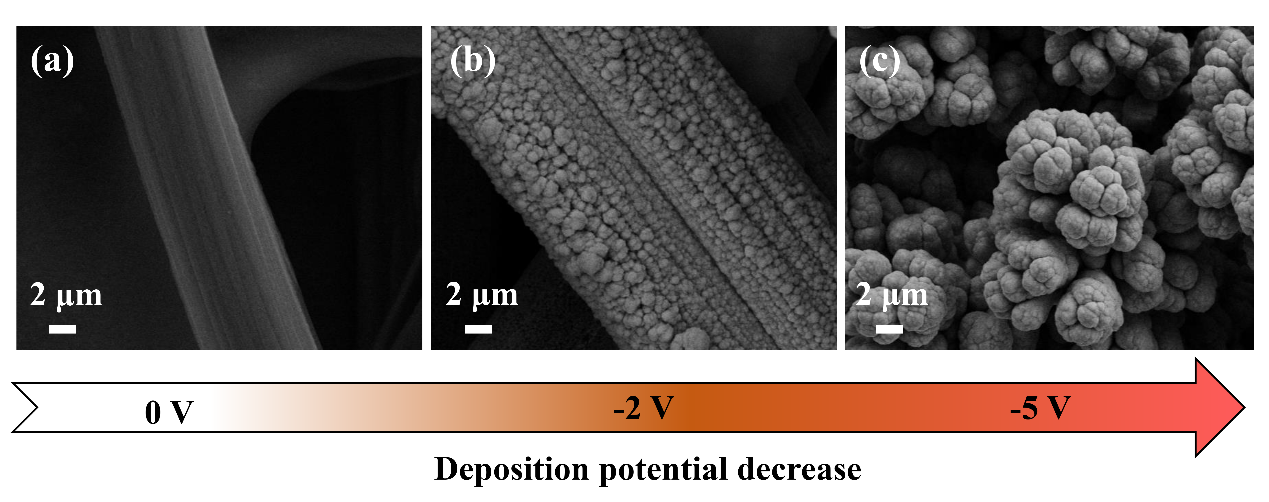


**Figure S3.** SEM image of deposited CoW with incremental current density (a) 0V (b) -2 V (c) -5 V.


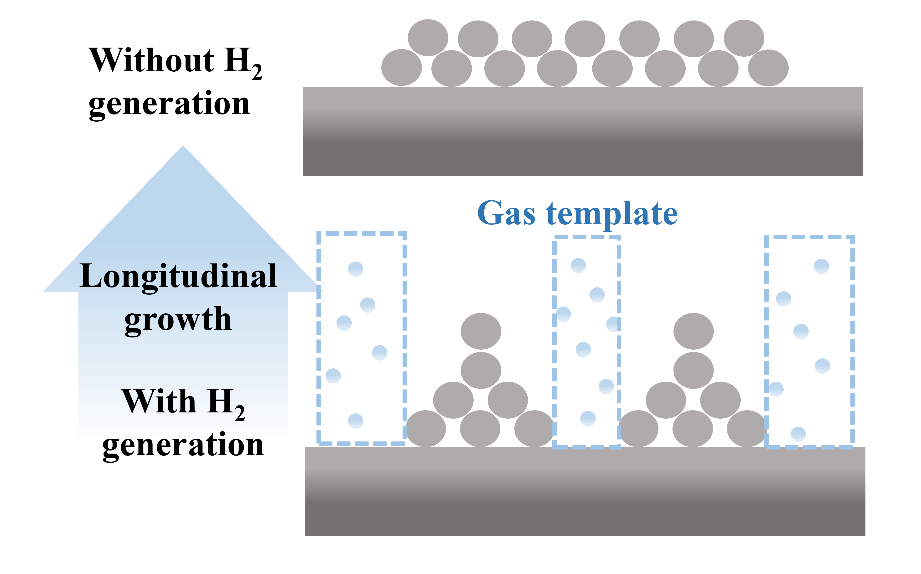


**Figure S4.** Graphically illustration of gas-template electrodeposition

With the decrease of the deposition potential, the deposition process is accompanied by more violent hydrogen evolution, which will induce the longitudinal growth of the sedimentary layer


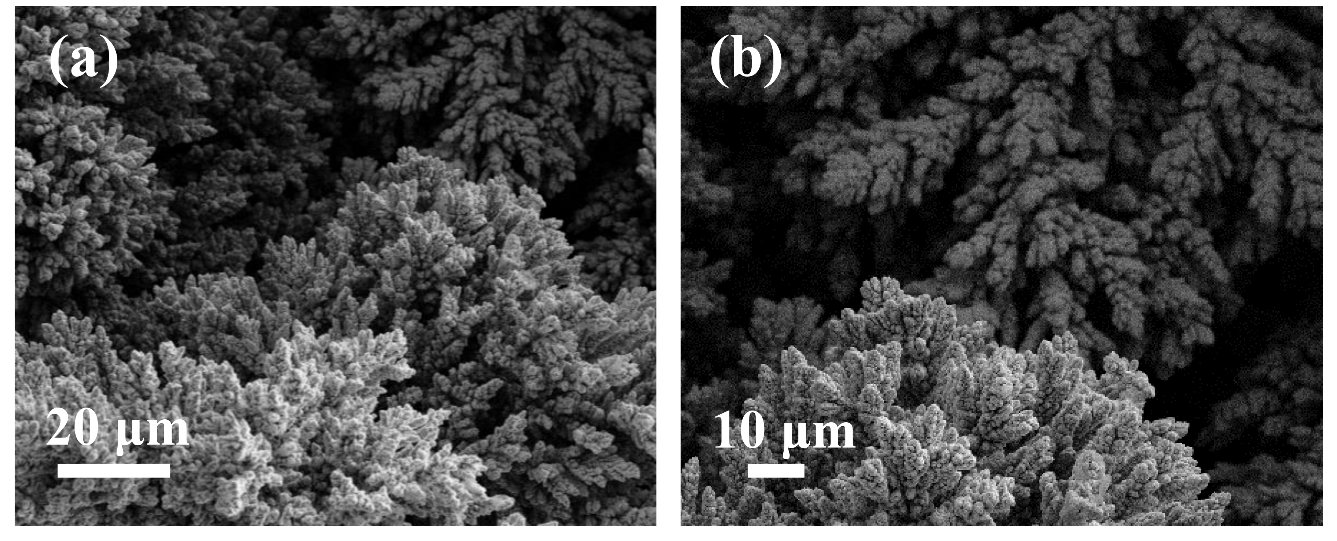


**Figure S5.** SEM image with low magnification of the deposited CoW at -8 V.


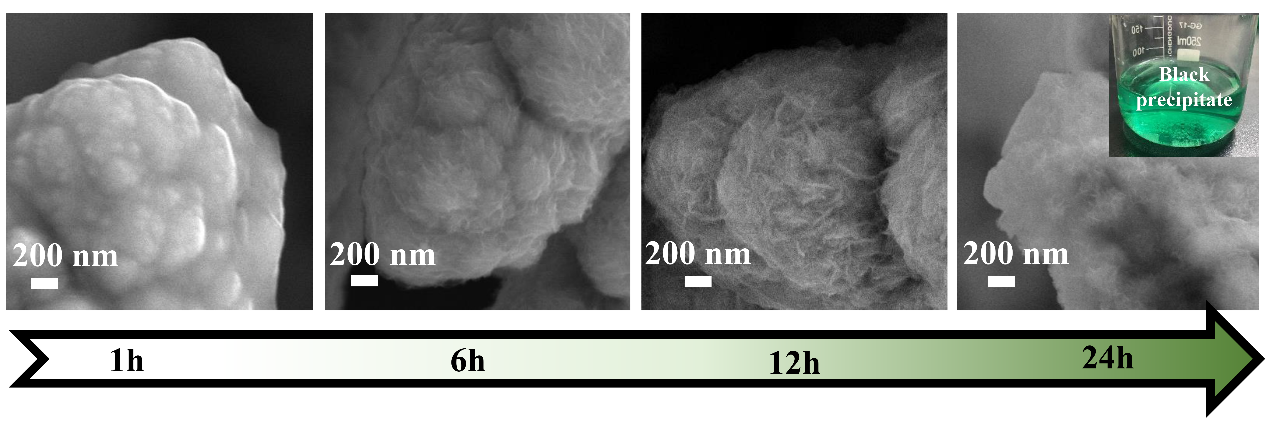


**Figure S6.** SEM image of as-prepared CoW-500-Ni with different soaking time (1, 6, 12 and 24h).

The surface appears covered with a flat topography at the initial stage of growth. The small size of the nanosheets grows in about 6h, and the size becomes larger with time of 12h. However, the morphology of the nanosheets disappears and the active material falls off in the solution after 24h. This is due to the destruction of the overall structure caused by prolonged soaking.





**Figure S7.** N_2_ absorption/desorption curves of CoW-500-Ni.


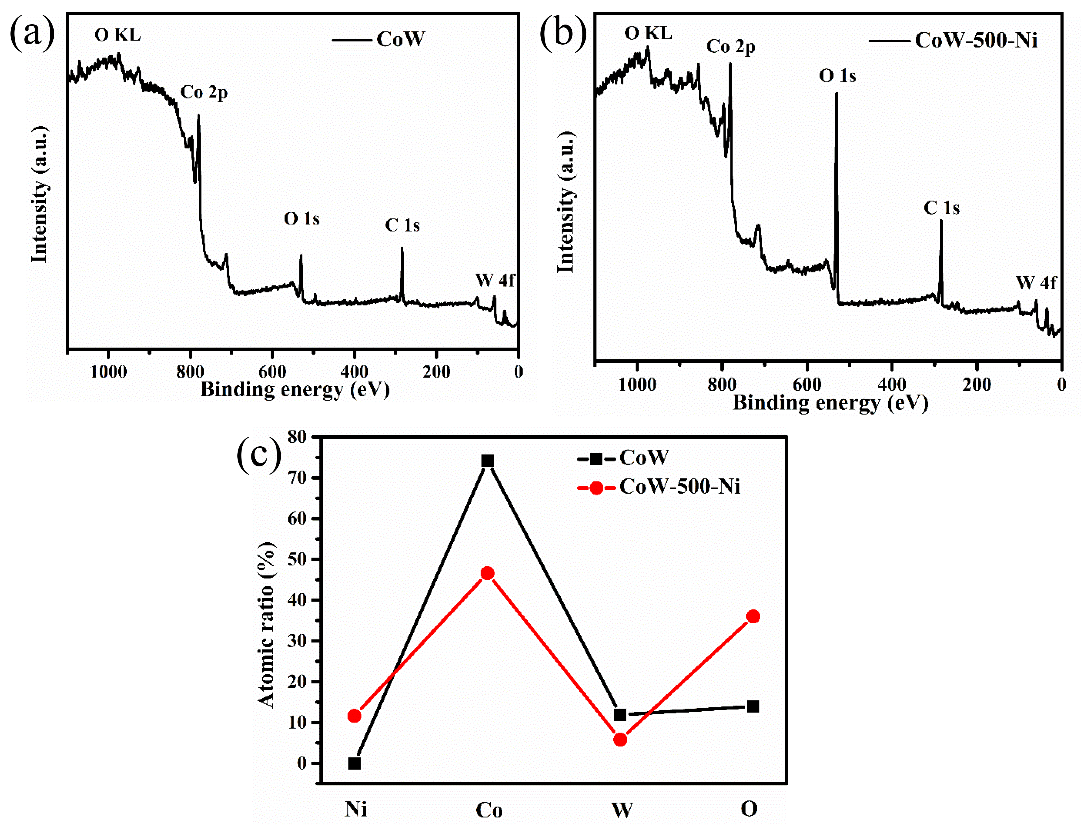


**Figure S8.** XPS survey spectra of (a) CoW and (b) CoW-500-Ni. (c) The element ratio of two group.


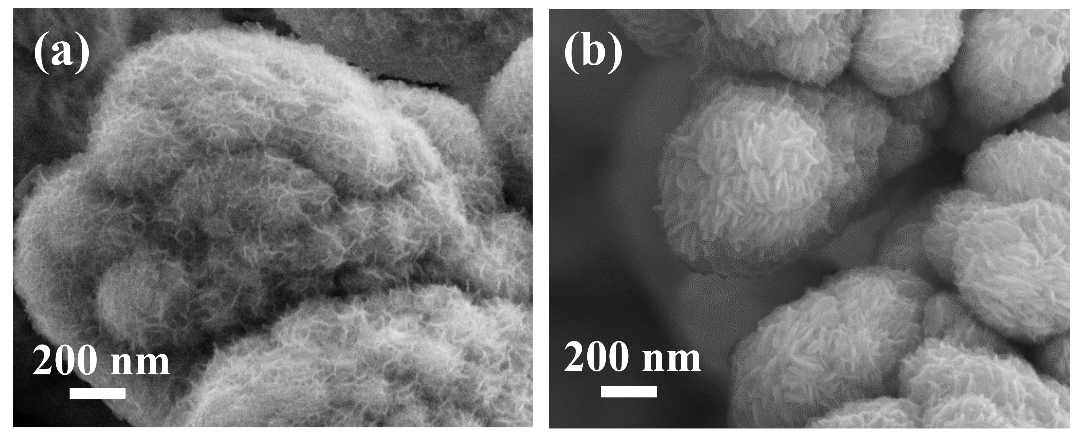


**Figure S9.** SEM images of all-prepared samples (a) CoW-0-Ni; (b) CoW-1000-Ni.

SEM exhibit the thickness of nanometer sheet changes regularly.


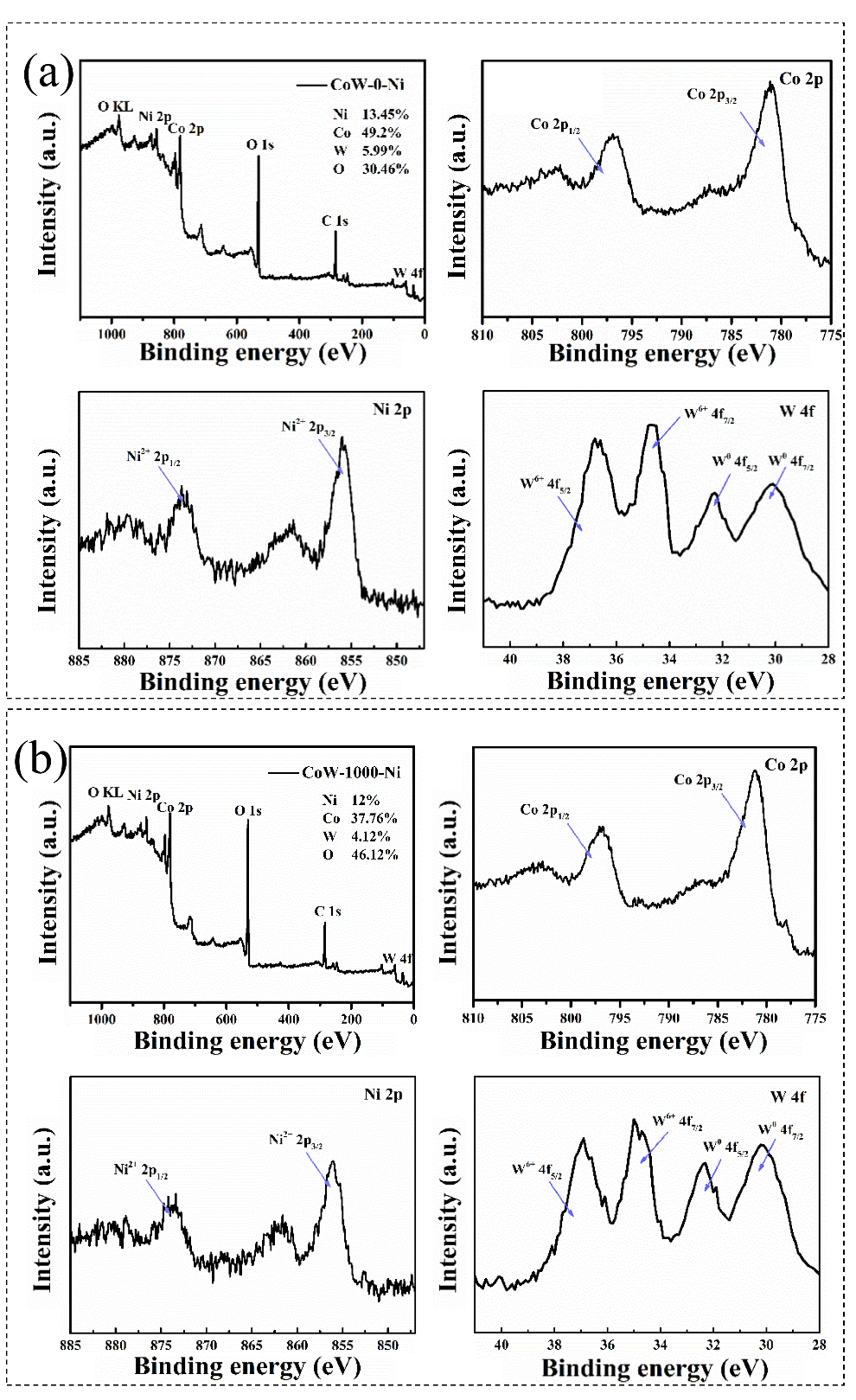


**Figure S10.** XPS spectra of (a) CoW-0-Ni; (b) CoW-1000-Ni.

XPS results confirm the atomic ratio between Ni, Co, W is basically flat and accompanied by the difference ratio of O.





**Figure S11.** Corrosion curve of CoW in different NaCl solution.

With the increase of Cl^-^ concentration, the corrosion current increased, indicating stronger corrosion behavior. Moreover, the comparison between Co and CoW solid solution in the same etching condition showed that the corrosion potential was almost equal, confirming that Co was involved in the reaction in the etching process.

**

**

**Figure S12.** XRD patterns of the deposited CoW and CoW-0-Ni, CoW-500-Ni, CoW-1000-Ni.


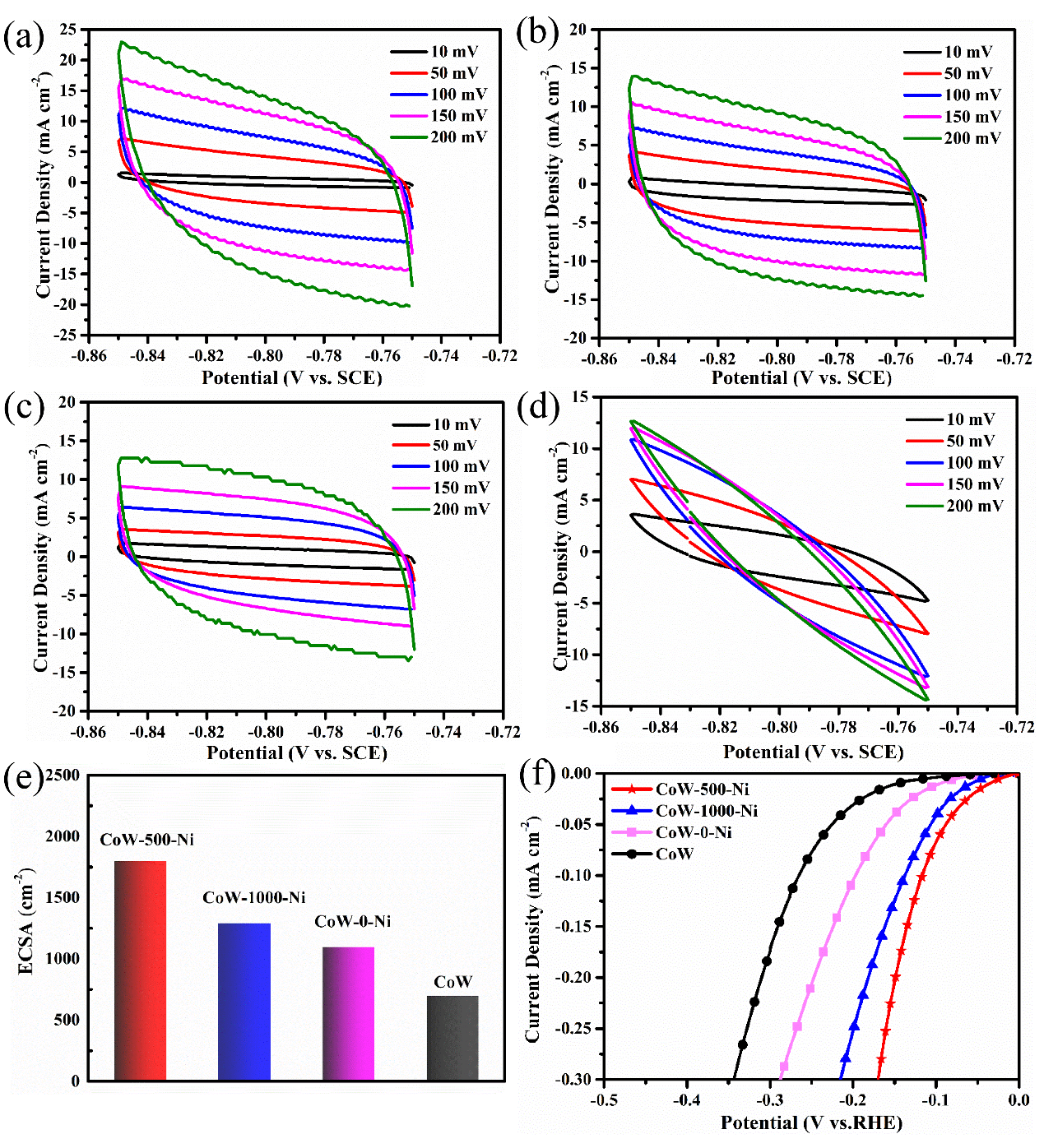


**Figure S13.** (a-d) CV curves of CoW-500-Ni, CoW-1000-Ni, CoW-0-Ni and CoW in 1M KOH solution. The scan rates from 10 mV s-1 to 200 mV s-1. (e) calculated ECSA values and (f) ECSA normalized LSV curves of all-prepared samples.

**

**

**Figure S14.** TOF normalized LSV curves of all-prepared samples.

TOF values were calculated by the formula: TOF = j × S /(2 × F × n), where j, S, F correspond to current density, geometric area and Faraday’s constant (96,485.3 C mol^-1^) respectively. n is the molar amount of all metals that assuming every metal atom is involved in the catalysis [15].

**
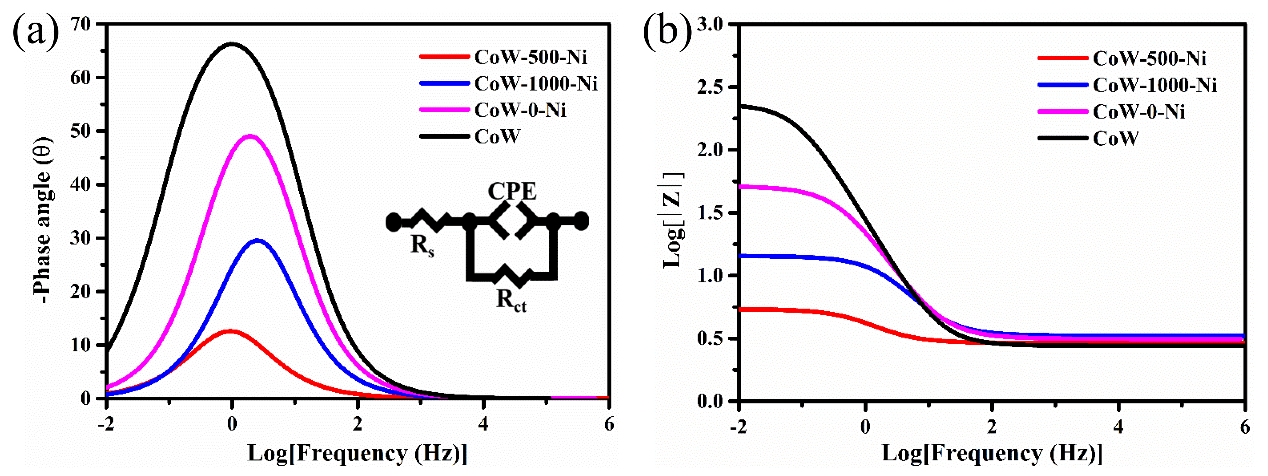
**

**Figure S15.** Bode plots of all-prepared samples correspond to Nyquist curves in Figure 5e.





**Figure S16.** The Faradaic efficiency (FE) of CoW-500-Ni in the HER process.


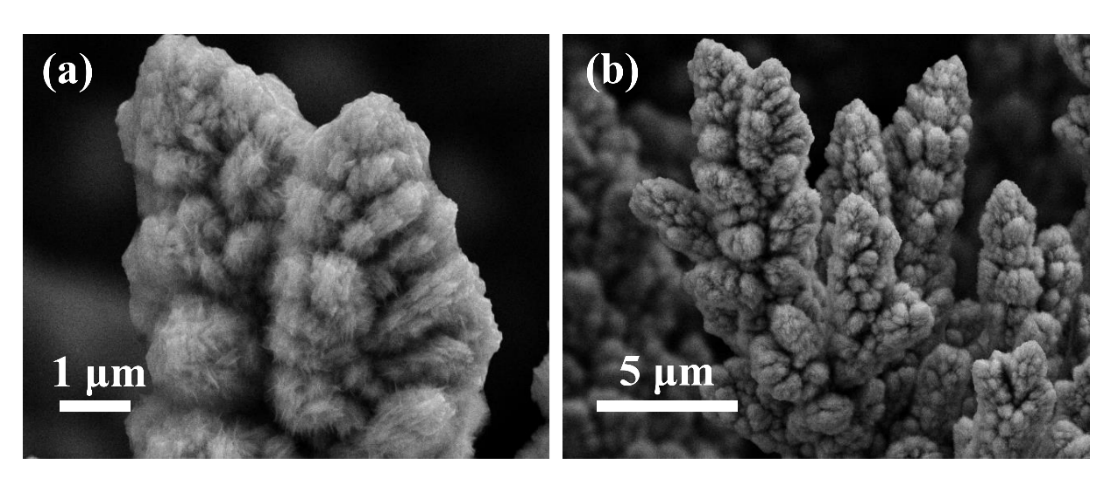


**Figure S17**. SEM images after HER test at 10 mA cm^-2^ with different magnification.


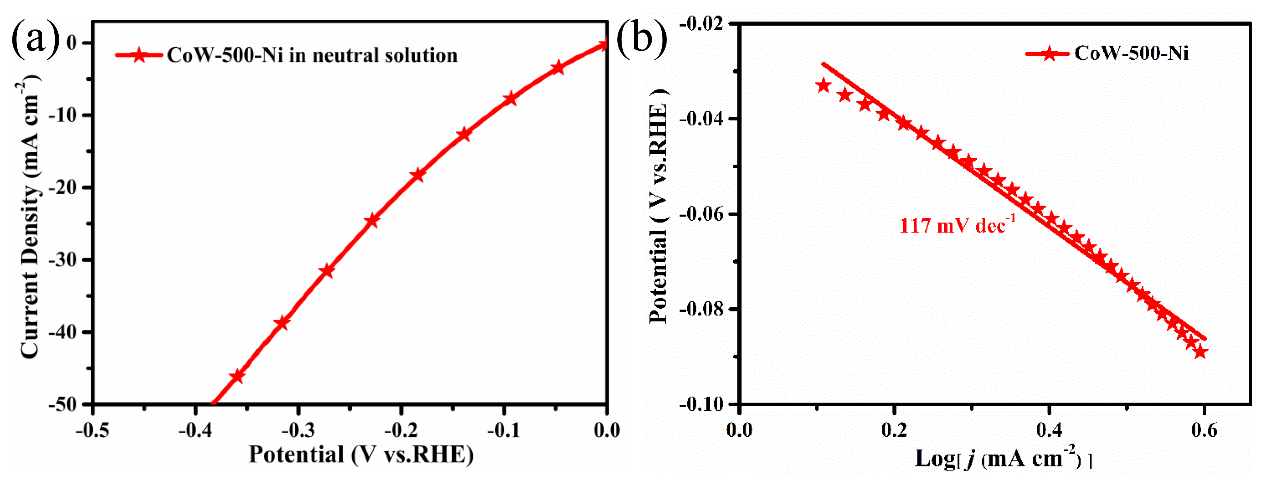


**Figure S18.** a) LSV curves and b) Tafel slope of CoW-500-Ni in neutral solution.





**Figure S19.** The OER performance of CoW, CoW-0-Ni and CoW-1000-Ni.





**Figure S20.** LSV curves of CoW-500-Ni with or without 0.3 M urea.


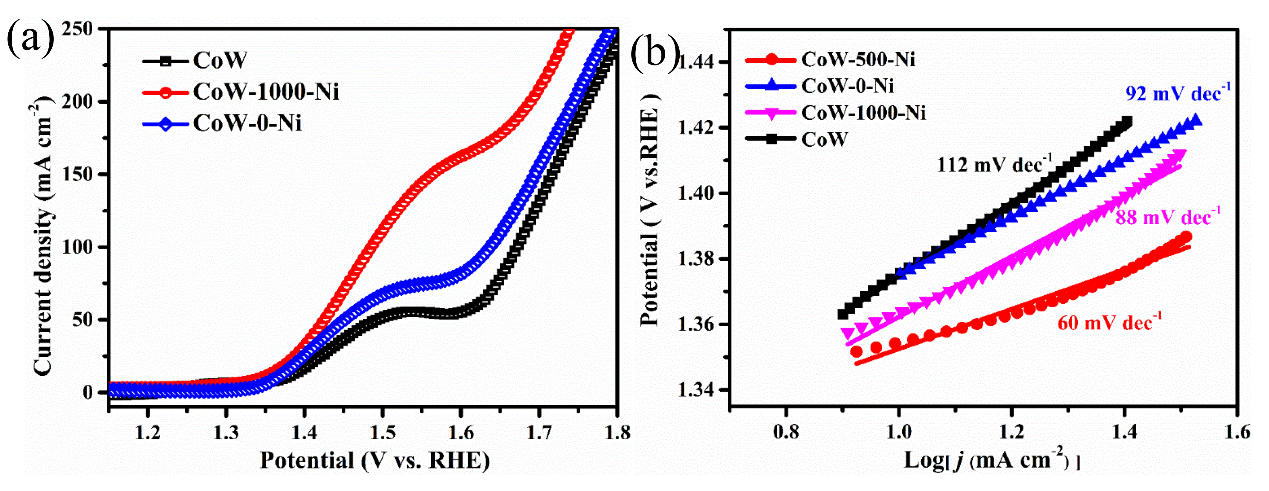


**Figure S21.** LSV curves of CoW, CoW-0-Ni and CoW-1000-Ni exhibit UOR performance (1 M KOH+ 0.3 M urea).





**Figure S22.** Comparison of the UOR performance of CoW-500-Ni with different urea concentration (0.1 and 0.5M urea).

The catalytic activity under low concentration of urea (0.1 M) is obviously poor. Increasing the concentration activity is significantly improved, and 0.3 M is suitable for its excellent catalytic performance and close to the concentration of domestic sewage, which increases its application significance.


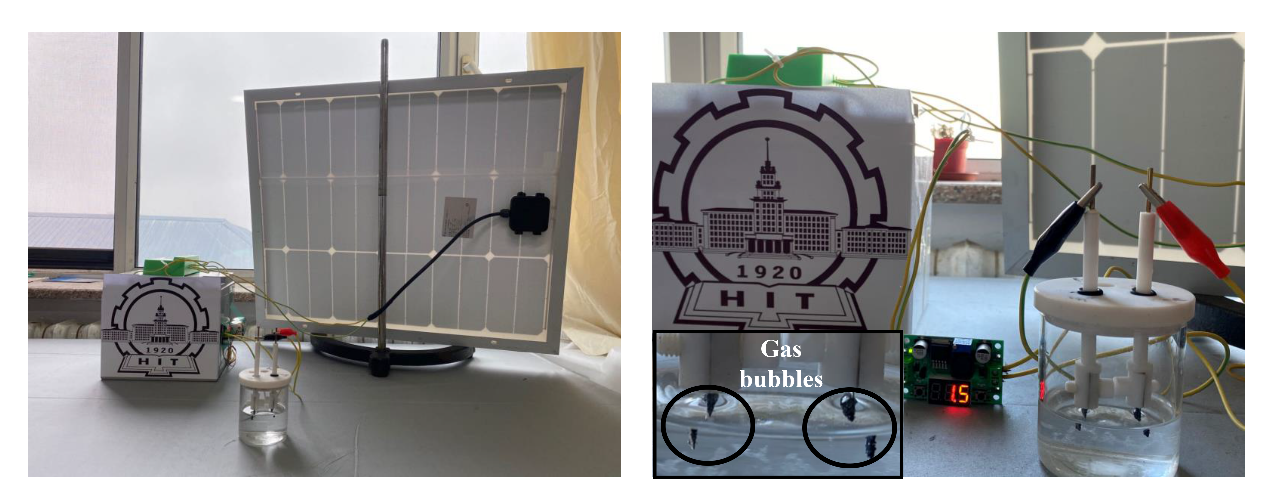


**Figure S23.** Photographic image of the solar-driven electrolysis system.

High efficiency hydrogen precipitation and urea oxidation reactions were simultaneously driven at a low potential of 1.5V.


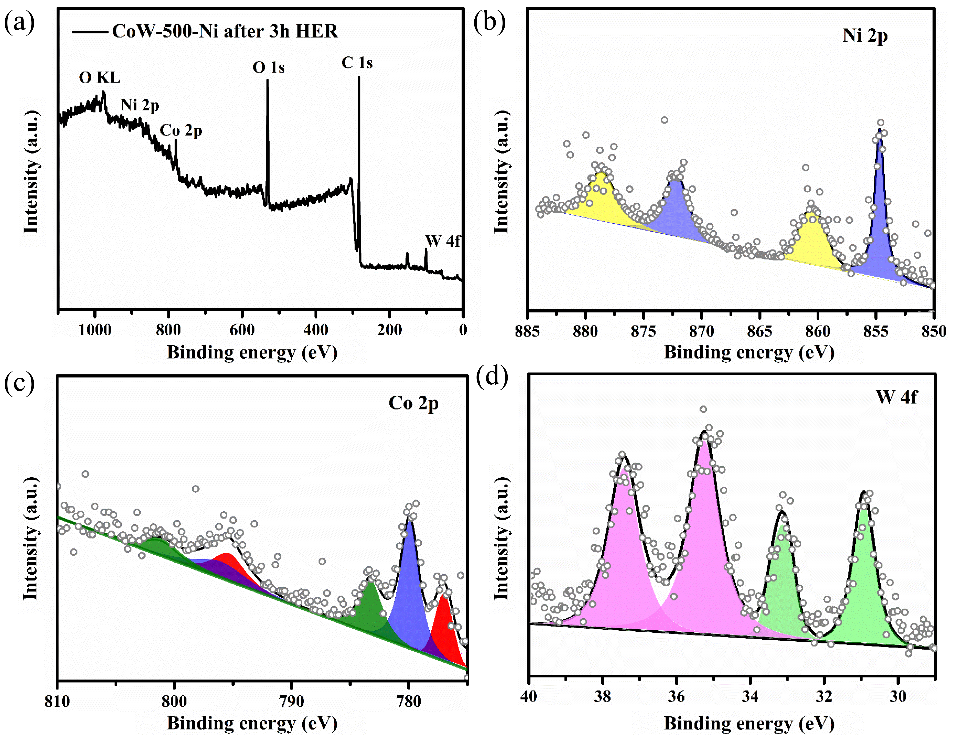


**Figure S24.** XPS spectra of CoW-500-Ni after 3h HER a) wide ranges spectra b) Ni 2p c) Co 2p and d) W 4f.


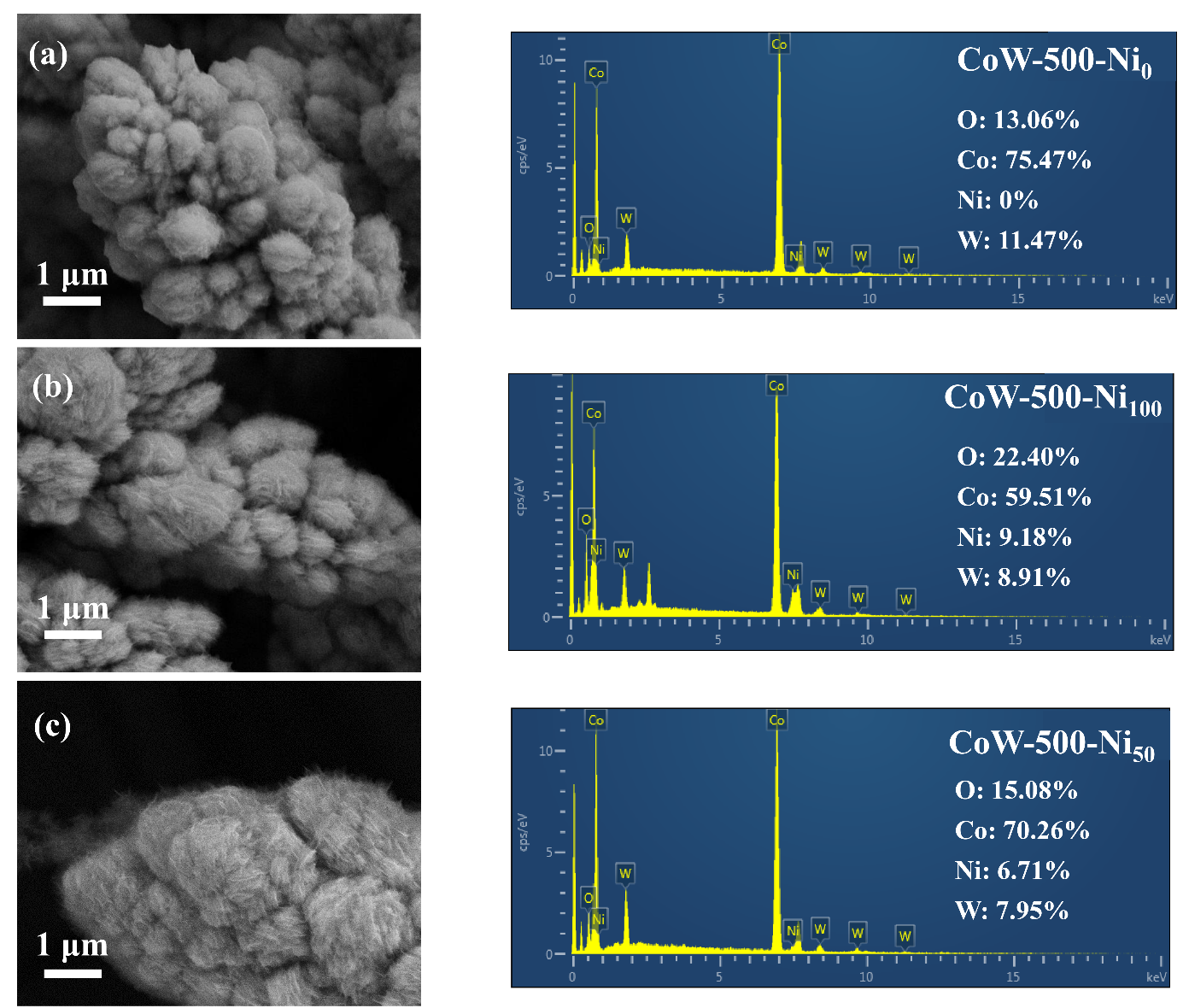


**Figure S25.** SEM EDS spectra for CoW-500-Ni with different concentration of Ni source a) 0 mM b) 100 mM c) 50 mM.





**Figure S26.** LSV curves of CoW-500-Ni_0_, CoW-500-Ni_50_ and CoW-500-Ni_100_.


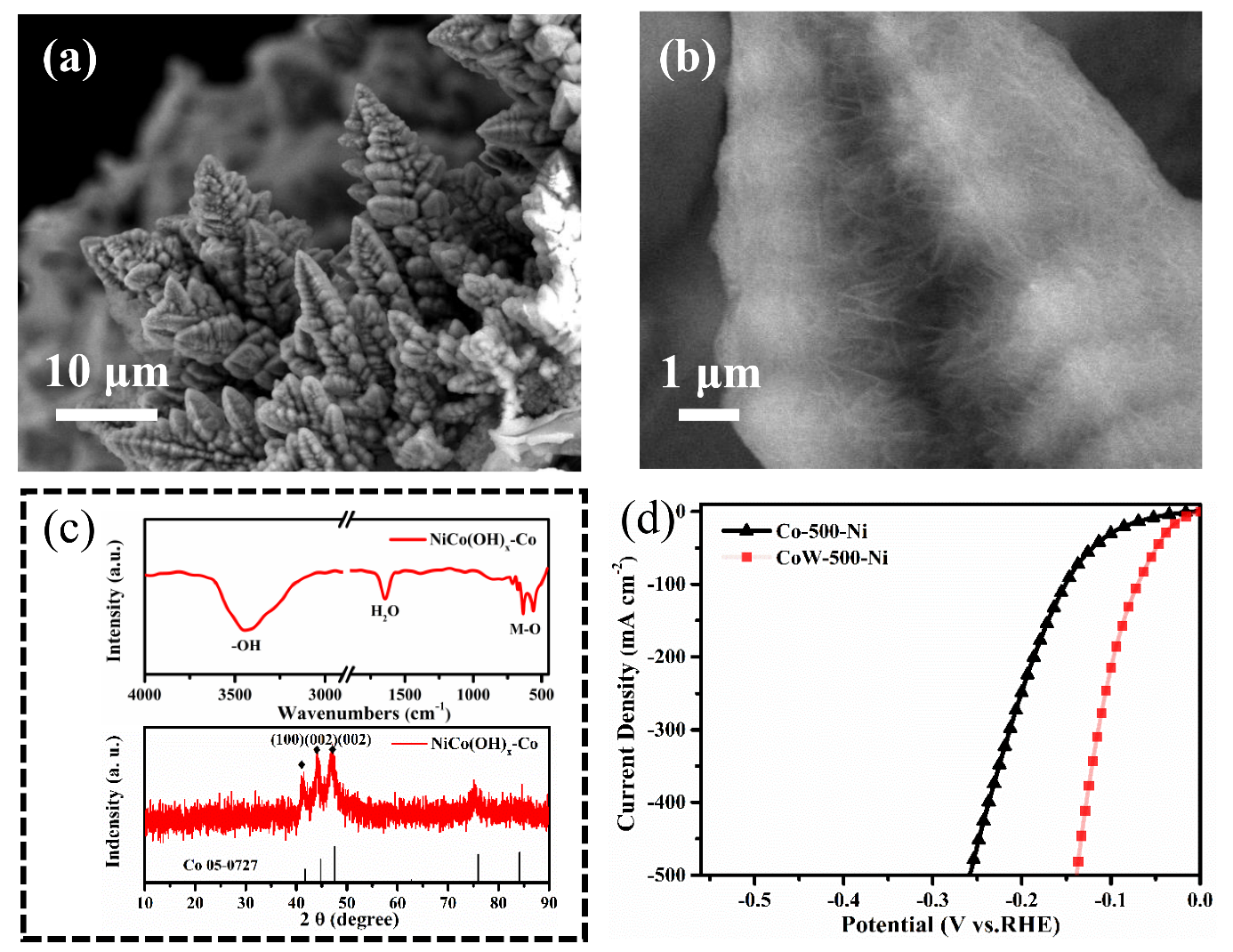


**Figure S27.** a, b) SEM images of Co-500-Ni with different magnification. c) FT-IR and XRD spectra of Co-500-Ni. d) LSV curve compared with CoW-500-Ni.

NiCo(OH)_x_-Co was synthesized by removing tungsten only with the same optimal preparation process. The coverage of hydroxide nanosheets on the surface can be observed in SEM image. XRD and FT-IR together confirm the same mixed-crystalline heterostructure as NiCo(OH)_x_-Co_y_W. In Figure S27d, the poor catalytic activity of NiCo(OH)_x_-Co (labled as Co-500-Ni) confirms the key role of the introduction of tungsten.

**Table S1.** ICP results of CoW, CoW-0-Ni, CoW-500-Ni and CoW-1000-Ni.

|  | **Co wt% (at %)** | **W wt% (at %)** | **Ni wt% (at %)** |
| --- | --- | --- | --- |
| **CoW** ^a)^ | 73.85 (90.7) | 23.27 (9.3) | 0 (0) |
| **CoW-0-Ni** ^b)^ | 75.43 (88.0) | 17.85 (6.8) | 4.26 (5.2) |
| **CoW-500-Ni** ^c)^ | 74.74 (87.5) | 17.46 (6.7) | 4.76 (5.8) |
| **CoW-1000-Ni** ^d)^ | 74.28 (87.1) | - 1. (6.5) | 5.30 (6.4) |

*Sample quality: a) 48.9 mg; b) 44.7 mg; c) 51.8 mg; d) 60.1 mg.

Fixed volume 50ml, diluted 50 times

**Table S2.** XPS analysis corresponding to Figure 4a-c.

| **Sample** | **B. E. (eV)** | **Peak** |
| --- | --- | --- |
| **CoW** | 873.4 | Ni2p _1/2_ |
|  | 855.8 | Ni2p _3/2_ |
|  | 796.1 | Co2p _1/2_ |
|  | 783.2 | Co^3+^ |
|  | 780.7 | Co2p _3/2_ |
|  | 773.6 | Co^0^ |
|  | 34.9 | W^6+^ |
|  | 30.8 | W^0^ |
| **CoW-500-Ni** | 795.8. | Co2p _1/2_ |
|  | 780.5 | Co2p _3/2_ |
|  | 777.1 | Co |
|  | 34.7 | W^6+^ |
|  | 30.6 | W^0^ |

**Table S3.** The preparation method, morphology, overpotential and Tafel slope of the non-noble metal-based HER electrocatalysts corresponding to Figure 5c.

| Non-noble metal-based HER electrocatalyst | Preparation method | Morphology | Overpotential  -10 mA cm^-2^  (mV) | Tafel slope  (mV dec^-1^) |
| --- | --- | --- | --- | --- |
| CoMn/CoMn_2_O_4_ [1] | Hydrothermal-annealing + electrochemical in situ tuning treatment | 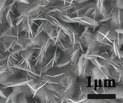 | 69 mV | 90 |
| Ni_5_Co_3_Mo−OH [2] | Chloride corrosion | 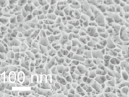 | 52 | 59 |
| Co_1−x_Ni_x_P_3_ [3] | Hydrothermal procedure + high- temperature  phosphidation | 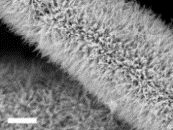 | 57 | 60.7 |
| V-Ni_3_S_2_@NiFe LDH [4] | Sulfuration V doping + electrodeposition of NiFe LDH | 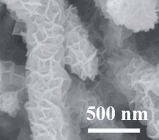 | 120 | 72.8 |
| Ni_3_N-VN [5] | Hydrothermal procedure + high- temperature  nitridaton | 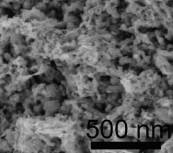 | 64 | 37 |
| P-Fe_3_N@NC NSs/IF [6] | Air-oxidation + high- temperature  nitridaton | 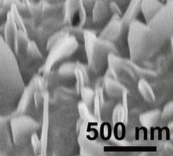 | 102 | 68.59 |
| Ni(OH)_2_-NiMoO_x_/NF [7] | ZnO-templated  electrodeposition + soaking in 1M KOH | 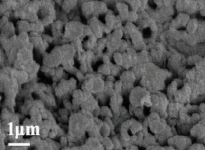 | 36 | 38 |
| V-CoP@a-CeO_2_ [8] | Hydrothermal reaction + phosphating and electrodeposition | 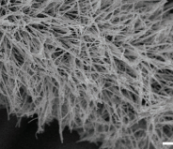 | 68 | 48.1 |
| CoNiP@CN [9] | Two-step electrodeposition mixed-metal ZIFs@ZIF-8 + pyrolysis and phosphorization | 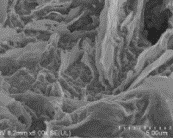 | 87 | 79 |
| Ni-S-P NRs/NF [10] | Phosphorization and sulfurization thermal treatment | 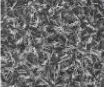 | 115 | 64.2 |
| Ni_17_W_3_/WO_2_/NF [11] | Precipitation reaction + calcination at 600℃ | 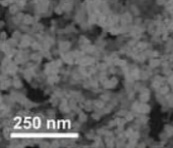 | 59 | 52 |
| Ni_2_P/NiTe_2_ [12] | Hydrothermal reaction + phosphorization | 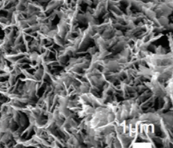 | 62 | 80 |
| TiO_2_@Ni_3_S_2_ [13] | Hydrothermal process + immersing them into a Na_2_S solution and heated | 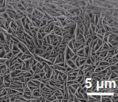 | 112 | 69 |
| NiCoFe-PS [14] | Novel hydrothermal electrodeposition + electrochemical dealloy and sulfidization / phosphorization | 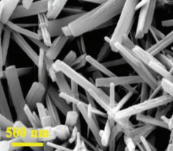 | 97.8 | 51.8 |

**Table S4.** Nyquist plot fitting results corresponding to Figure 5e.

|  | **Rs** | **Rct** | **CPE1-T** | **CPE1-P** |
| --- | --- | --- | --- | --- |
| **CoW-500-Ni** | 2.89 | 2.54 | 0.13 | 0.77 |
| **CoW-1000-Ni** | 3.22 | 11.29 | 0.011 | 0.79 |
| **CoW-0-Ni** | 3.26 | 41.03 | 0.008 | 0.86 |
| **CoW** | 2.62 | 203.8 | 0.015 | 0.87 |

**References**

1. C. Wang, H. L. Lu, Z. Y. Mao, C. L. Yan, G. Z. Shen, X. F. Wang. Bimetal schottky heterojunction boosting energy-saving hydrogen production from alkaline water via urea electrocatalysis. Adv Funct Mater. **30**(21), 10 (2020). <https://doi.org/10.1002/adfm.202000556>
2. S. Y. Hao, L. C. Chen, C. L. Yu, B. Yang, Z. J. Li, Y. Hou, L. C. Lei, X. W. Zhang. Nicomo hydroxide nanosheet arrays synthesized via chloride corrosion for overall water splitting. ACS Energy Lett. **4**(4), 952-959 (2019).

<https://doi.org/10.1021/acsenergylett.9b00333>

1. Q. Fu, T. Wu, G. Fu, T. L. Gao, J. C. Han, T. Yao, Y. M. Zhang, W. W. Zhong, X. J. Wang, B. Song. Skutterudite-type ternary co1-xnixp3 nanoneedle array electrocatalysts for enhanced hydrogen and oxygen evolution. ACS Energy Lett. **3**(7), 1744-1752 (2018).

<https://doi.org/10.1021/acsenergylett.8b00908>

1. J. Q. Zhou, L. Yu, Q. C. Zhu, C. Q. Huang, Y. Yu. Defective and ultrathin nife ldh nanosheets decorated on v-doped ni3s2 nanorod arrays: A 3d core-shell electrocatalyst for efficient water oxidation. J Mater Chem A. **7**(30), 18118-18125 (2019).

<https://doi.org/10.1039/c9ta06347a>

1. H. J. Yan, Y. Xie, A. P. Wu, Z. C. Cai, L. Wang, C. G. Tian, X. M. Zhang, H. G. Fu. Anion-modulated her and oer activities of 3d ni-v-based interstitial compound heterojunctions for high-efficiency and stable overall water splitting. Adv Mater. **31**(23), 9 (2019). <https://doi.org/10.1002/adma.201901174>
2. G. X. Li, J. Y. Yu, W. Q. Yu, L. J. Yang, X. L. Zhang, X. Y. Liu, H. Liu, W. J. Zhou. Phosphorus-doped iron nitride nanoparticles encapsulated by nitrogen-doped carbon nanosheets on iron foam in situ derived fromsaccharomycetes cerevisiaefor electrocatalytic overall water splitting. Small. **16**(32), 11 (2020).

<https://doi.org/10.1002/smll.202001980>

1. Z. H. Dong, F. Lin, Y. H. Yao, L. F. Jiao. Crystalline ni(oh)(2)/amorphous nimoox mixed-catalyst with pt-like performance for hydrogen production. Adv Energy Mater. **9**(46), 7 (2019).

<https://doi.org/10.1002/aenm.201902703>

1. L. Yang, R. M. Liu, L. F. Jiao. Electronic redistribution: Construction and modulation of interface engineering on cop for enhancing overall water splitting. Adv Funct Mater. **30**(14), 8 (2020).

<https://doi.org/10.1002/adfm.201909618>

1. L. J. Yang, L. Zhang. N-enriched porous carbon encapsulated bimetallic phosphides with hierarchical structure derived from controlled electrodepositing multilayer zifs for electrochemical overall water splitting. Appl Catal B-Environ. **259**(9 (2019). <https://doi.org/10.1016/j.apcatb.2019.118053>
2. Y. Zhou, T. T. Li, S. Q. Xi, C. He, X. G. Yang, H. J. Wu. One-step synthesis of self-standing ni3s2/ni2p heteronanorods on nickel foam for efficient electrocatalytic hydrogen evolution over a wide ph range. ChemCatChem. **10**(23), 5487-5495 (2018). <https://doi.org/10.1002/cctc.201801373>
3. C. Han, D. W. Wang, Q. Li, Z. C. Xing, X. R. Yang. Ni17w3 nanoparticles decorated wo2 nanohybrid electrocatalyst for highly efficient hydrogen evolution reaction. ACS Appl Energ Mater. **2**(4), 2409-2413 (2019).

<https://doi.org/10.1021/acsaem.9b00170>

1. Y. B. Li, X. Tan, H. Tan, H. J. Ren, S. Chen, W. F. Yang, S. C. Smith, C. Zhao. Phosphine vapor-assisted construction of heterostructured ni2p/nite(2)catalysts for efficient hydrogen evolution. Energy Environ Sci. **13**(6), 1799-1807 (2020). <https://doi.org/10.1039/d0ee00666a>
2. S. J. Deng, K. L. Zhang, D. Xie, Y. Zhang, Y. Q. Zhang, Y. D. Wang, J. B. Wu, X. L. Wang, H. J. Fan, X. H. Xia, J. P. Tu. High-index-faceted ni3s2 branch arrays as bifunctional electrocatalysts for efficient water splitting. Nano-Micro Lett. **11**(1), 12 (2019). <https://doi.org/10.1007/s40820-019-0242-8>
3. M. Q. Yao, H. H. Hu, B. L. Sun, N. Wang, W. C. Hu, S. Komarneni. Self-supportive mesoporous Ni/Co/Fe phosphosulfide nanorods derived from novel hydrothermal electrodeposition as a highly efficient electrocatalyst for overall water splitting. Small. **15**(50), 9 (2019).

<https://doi.org/10.1002/smll.201905201>

1. P. T. Liu, J. Q. Ran, B. R. Xia, S. B. Xi, D. Q. Gao, John Wang. Bifunctional Oxygen Electrocatalyst of Mesoporous Ni/NiO Nanosheets for Flexible Rechargeable Zn–Air Batteries. Nano-Micro Lett. **68**(1), 12 (2020).

<https://doi.org/10.1007/s40820-020-0406-6>
